# Associations Between Prepregnancy Menstrual Characteristics, Age at Menarche, and the Risk of Gestational Diabetes Mellitus: A Matched Case–Control Study

**DOI:** 10.1155/jdr/2596620

**Published:** 2026-01-22

**Authors:** Yushuang Su, Qin Yang, Cui Xing, Hui Wang, Rong Li, Juan Zhang, Jie Mei, Jing He

**Affiliations:** ^1^ Department of Thoracic Surgery, Sichuan Provincial People′s Hospital, University of Electronic Science and Technology of China, Chengdu, China, uestc.edu.cn; ^2^ Department of Nursing, Sichuan Provincial People′s Hospital, University of Electronic Science and Technology of China, Chengdu, China, uestc.edu.cn; ^3^ Department of Nursing, Tongji Hospital, Tongji Medical College, Huazhong University of Science and Technology, Wuhan, China, hust.edu.cn; ^4^ Department of Obstetrics and Gynecology, Sichuan Provincial People′s Hospital, University of Electronic Science and Technology of China, Chengdu, China, uestc.edu.cn

**Keywords:** gestational diabetes mellitus, menarche, menstrual cycle, pregnancy

## Abstract

**Background:**

Inconsistencies exist in the literature regarding the associations among age at menarche (AAM), prepregnancy menstrual characteristics, and the risk of gestational diabetes mellitus (GDM). These discrepancies may be attributable to variations in population demographics. The aim of this study was to investigate the impact of prepregnancy menstrual characteristics and AAM on the likelihood of developing GDM among Chinese women.

**Methods:**

A 1:1 age‐matched case–control study was conducted that included 2289 patients with GDM and 2289 normoglycemic pregnant women as controls at Wuhan Union Hospital from September 2020 to August 2022. Fasting blood samples were collected during 24–28 weeks of gestation. AAM and menstrual cycle characteristics were categorized and incorporated into a conditional logistic regression model that was adjusted for potential confounders. Additionally, restricted cubic spline curves were employed to assess the trend in GDM risk associated with AAM.

**Results:**

The final analysis included 4578 participants. The AAM in the GDM group presented significantly earlier than that in the normoglycemic group (*p* < 0.05). After adjusting for confounding factors, we found that women with an AAM of 12 years (aOR = 1.44, 95% CI: 1.25–1.67) or 14 years (aOR = 1.36, 95% CI: 1.15–1.61) had a significantly higher risk of developing GDM compared with those with an AAM of 13 years. Furthermore, analysis of the data by means of restricted cubic splines revealed an L‐shaped association that linked AAM to GDM (*p* < 0.001). The association between prolonged and irregular menstrual cycles and GDM risk remained statistically significant, albeit attenuated, after multivariable adjustment. Irregular menstrual cycles (classified as “usually irregular” or “always irregular”) were significantly associated with an increased risk of GDM, with aORs of 2.36 (95% CI: 1.47–3.79) and 2.40 (95% CI: 1.01–5.71), respectively. Moreover, menstrual cycle durations of 32–39 days or more than 50 days were significantly associated with an increased risk of GDM (aORs: 1.20 and 1.37; 95% CIs: 1.10–1.41 and 1.03–1.83, respectively).

**Conclusion:**

Early AAM, irregular menstrual cycles, and prolonged menstrual cycle length were associated with an increased risk of GDM. Among women with menarche occurring before the age of 13, there was an association with a higher risk of GDM. These indicators may help identify women at high risk and facilitate preconception interventions to prevent GDM.

**Trial Registration:**

ClinicalTrials.gov identifier: ChiCTR2200063189

## 1. Background

Gestational diabetes mellitus (GDM) is a metabolic disorder unique to pregnancy, typically identified in the later stages of gestation, that is characterized by impaired glucose tolerance [1]. As one of the most common obstetric complications globally, the incidence of GDM varies significantly across populations due to divergent diagnostic criteria and demographic profiles [2]. According to the diagnostic guidelines established by the International Association of Diabetes and Pregnancy Study Groups (IADPSG), the prevalence of GDM in China is estimated to be between 14.8% and 19.7% [3, 4]. The clinical significance of GDM extends beyond pregnancy, with approximately 20% of affected women progressing to Type 2 diabetes mellitus (T2DM) within 5–10 years postpartum [5, 6]. Additionally, GDM is associated with adverse maternal outcomes (e.g., preterm birth and cesarean delivery) and long‐term offspring health risks, including neonatal jaundice, childhood obesity, and metabolic syndrome [7–9]. The health and economic benefits of early diagnosis and intervention for women at risk of GDM are substantial [10].

Menarche marks the onset of the first menstruation and the initiation of the ovarian and endocrine functions related to reproduction. Age at menarche (AAM) is determined by the interplay between genetic and environmental factors. Early AAM has been associated with an increased risk of T2DM, cardiovascular diseases, and metabolic syndrome [11, 12]. However, inconsistent associations between AAM and the risk of GDM during pregnancy have been reported [13–15]. For example, research from Japan [16] suggested that females who experience AAM ≤ 9 years have an elevated risk of GDM, whereas a study from Turkey [17] identified AAM < 12 years as a predictor of GDM; in contrast, studies conducted in the United States reported no significant association between AAM and GDM risk [13]. Menstrual cycles with irregular intervals and prolonged durations are common manifestations of endocrine disorders. Moreover, such menstrual irregularities, which reflect disturbances in the functional integrity of the hypothalamic–pituitary–ovarian axis, are associated with insulin resistance [18]. Given that insulin functions as both a reproductive hormone and a metabolic hormone [19], the potential relationships between AAM and menstrual characteristics and the risk of GDM warrant further investigation. The aim of this study was to elucidate the associations between AAM, prolonged or irregular menstrual cycles, and the risk of developing GDM. We hypothesized that early AAM and menstrual irregularities independently predict GDM risk.

## 2. Methods

### 2.1. Study Design and Population

This was a 1:1 age‐matched case–control study conducted at Wuhan Union Hospital in China. Pregnant women who received prenatal care and delivered at the hospital between September 2020 and August 2022 were identified from medical records for the study. All participants underwent an oral glucose tolerance test (OGTT) between 24 and 28 weeks of gestation. The study protocol was approved by the Ethics Committee of Wuhan Union Hospital, Huazhong University of Science and Technology (TJ‐IRB20220611) [20].

Participants were excluded from the study if they met any of the following criteria: (a) had multiple pregnancies, (b) had prepregnancy diabetes, (c) had pregnancies complicated by infectious diseases, or (d) had severe preexisting medical conditions that could significantly impact pregnancy outcomes, such as heart disease, immune system disorders, or mental illness.

### 2.2. Data Collection

Subsequently, data for the following variables were retrieved from the delivery records of the hospital′s information system: AAM, prepregnancy menstrual characteristics, maternal age, education, parity, gravidity, history of cesarean section, history of abnormal pregnancies, body mass index (BMI) before the first trimester, use of assisted reproductive technology (ART), and the presence of conditions such as GDM, gestational transient hyperthyroidism (GTH), hypothyroidism in pregnancy, thalassemia, polycystic ovary syndrome (PCOS), pregnancy‐induced hypertension (PIH), and Hashimoto′s thyroiditis (HT).

### 2.3. Definitions

The GDM of the subjects was evaluated via a 75‐g OGTT administered between 24 and 28 weeks of gestation. Pregnant women were diagnosed with GDM if any of the following plasma glucose thresholds were met or exceeded: 5.1 mmol/L at 0 h, 10.0 mmol/L at 1 h, or 8.5 mmol/L at 2 h following the 75‐g glucose load [21].

AAM was determined as the age at first menstruation and was categorized as follows: ≤ 11, 12, 13, 14, 15, and ≥ 16 years. In China, the average AAM for females born between 1973 and 2004 was approximately 13 years. Consequently, this age was adopted as the reference group for menarche in our study [22]. The cycle length was categorized as follows: ≤ 21, 21–25, 26–31, 32–39, 40–50, and > 50 days or too irregular to determine. Considering that the typical menstrual cycle for young, healthy women is 28 days (i.e., the interval from the first day of menstruation to the onset of the next cycle) [23] and acknowledging the considerable variability in cycle length among women, this study used a cycle length of 26–31 days as the reference group [24]. Cycle regularity was categorized as follows: very regular (3–4 days), regular (5–7 days), usually irregular, and always irregular; the regular category served as the reference group.

A history of abnormal pregnancy was defined as any prior adverse pregnancy outcome, including recurrent miscarriage (abortion ≥ 2 times), history of embryonic arrest, stillbirth, history of birth defects, and history of ectopic pregnancy [25, 26].

### 2.4. Data Analysis

Continuous variables are presented as the means ± standard deviations, whereas categorical variables are expressed as frequencies. Comparisons of normally distributed continuous variables between groups were performed via the independent‐samples *t* *-*test, whereas the Mann–Whitney *U* test was applied to nonnormally distributed continuous variables. The associations between categorical variables across groups were analyzed via the chi‐square test. Variables with less than 10% missing data were not subjected to any imputation. Regression models were adjusted for potential confounders, including education, parity [27], gravidity, history of abnormal pregnancy [28], ART, PIH, PCOS [29], GTH, and hypothyroidism, as these factors are well recognized as influencing GDM. The selection of these variables was guided by both their clinical relevance and their significant associations identified in the univariate analysis. Conditional logistic regression analysis was conducted to assess the risk of GDM in different AAM groups (≤ 11, 12, 13, 14, 15, and ≥ 16 years; the 13‐year group represented the median age and was set as the reference group). The characteristics of the menstrual cycle were analyzed via the same method. Two risk measures were calculated: the unadjusted odds ratio (OR), representing the crude association between exposure and outcome, and the adjusted odds ratio (aOR), reflecting the association after controlling for potential confounders. Furthermore, restricted cubic spline curves were used to assess the trend in the relationship between AAM and the risk of GDM. Statistical analyses were performed via Stata software (Version 17.0) and R (Version 4.4.1). All tests were two‐sided, and a *p* value of < 0.05 was considered statistically significant.

## 3. Results

**Figure 1:**
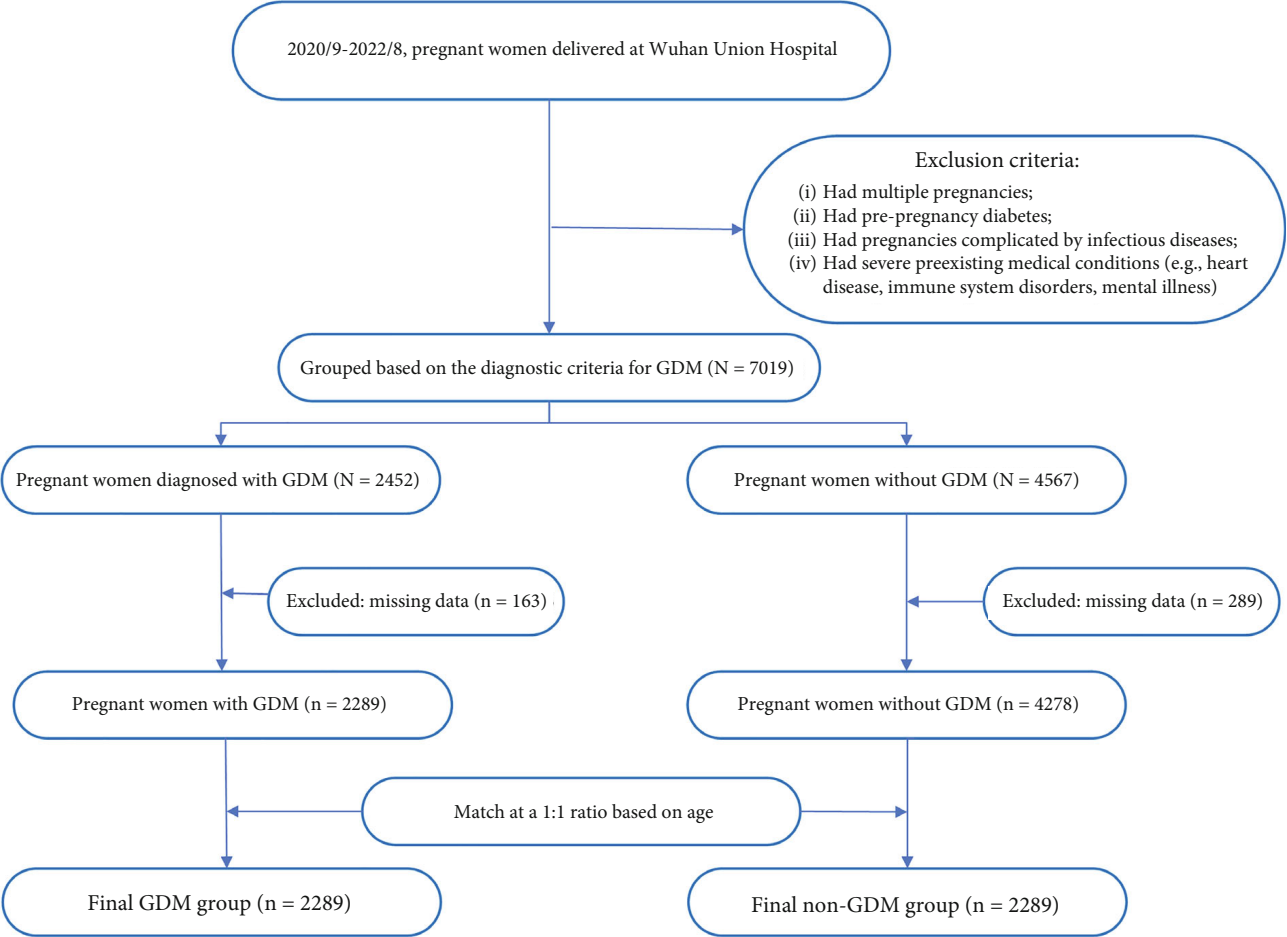
Flowchart of study participants.

According to the set inclusion criteria, 7019 pregnant women were initially enrolled. The study flowchart is presented in Figure 1. Of these, 2452 were diagnosed with GDM. After excluding 163 individuals with missing core data, 2289 women were included in the GDM group. Among the 4567 women with normal OGTT results, 289 were excluded due to missing data, resulting in 4278 eligible non‐GDM individuals. Subsequently, a random selection of 2289 women from this non‐GDM pool was performed to form the age‐matched (1:1) control group.

The average age of the 4578 participants was 30.69 ± 3.83 years. Table 1 compares demographic and clinical characteristics between 2289 GDM cases and 2289 controls. (The data summarized in Table 1 can be found at the end of the document [pages 26–27].) Furthermore, differences in AAM and menstrual characteristics between cases and controls were calculated. Compared with the control group, GDM patients presented with earlier AAM (12.90 ± 1.07 vs. 13.00 ± 1.10 years, *p* < 0.05). The prepregnancy BMI was similar between the groups (22.23 ± 3.51 vs. 22.13 ± 3.31, *p* = 0.300), indicating that there was no significant difference in the baseline BMI. In contrast, the distribution of education levels differed significantly between cases and controls (*p* < 0.001), and variations were observed across all categories. Although the prevalence of a family history of diabetes was slightly lower in cases (4.02%) than in controls (4.59%), this difference was not significant (*p* = 0.344). Furthermore, patients with GDM had significantly lower gravidity and parity compared with controls. The prevalence of a history of a cesarean section was marginally greater among the controls (22.41% vs. 20.05%, *p* = 0.051), although this difference did not reach statistical significance. A striking difference was observed in the prevalence of a history of an abnormal pregnancy, which was considerably greater in the GDM group (6.42% vs. 0.35%, *p* < 0.001). The use of ART was more prevalent among GDM cases (12.01% vs. 9.39%, *p* = 0.004). Additionally, PIH was significantly more common among GDM cases (2.80% vs. 0.44%, *p* < 0.001). Similar trends were observed for GTH (7.03% vs. 2.84%, *p* < 0.001), hypothyroidism during pregnancy (1.27% vs. 0.52%, *p* = 0.008), and PCOS (1.18% vs. 0.31%, *p* = 0.001).

**Table 1:** Demographic and baseline clinical characteristics of the case and control groups.

	**Case (** **n** = 2289**)**	**Control (** **n** = 2289**)**	**t**/**χ** ^2^	**p**
Age (years)	30.77 ± 3.68	30.61 ± 3.98	−1.38	0.167
AAM (years)			34.91	< 0.001
≤ 11	122 (5.33%)	109 (4.76%)		
12	767 (33.51%)	636 (27.79%)		
13	784 (34.25%)	937 (40.93%)		
14	478 (20.88%)	434 (18.96%)		
15	99 (4.33%)	110 (4.81%)		
≥ 16	39 (1.70%)	63 (2.75%)		
Menstrual cycle regularity			25.45	< 0.001
Most regular	264 (11.53%)	250 (10.92%)		
Regular	1931 (84.36%)	2001 (87.42%)		
Usually irregular	72 (3.15%)	30 (1.31%)		
Always irregular	22 (0.96%)	8 (0.35%)		
Menstrual cycle length (days)			14.27	0.014
< 21	71 (3.10%)	80 (3.49%)		
21–25	5 (0.13%)	6 (0.26%)		
26–31	1591 (69.51%)	1679 (73.35%)		
32–39	422 (18.44%)	360 (15.73%)		
40–50	67 (2.93%)	69 (3.01%)		
> 50	133 (5.81%)	95 (4.15%)		
Education			67.85	< 0.001
Elementary and below	205 (8.96%)	89 (3.89%)		
Middle school	201 (8.78%)	241 (10.53%)		
High school	222 (9.70%)	292 (12.76%)		
Junior college	597 (26.08%)	571 (24.95%)		
Undergraduate	840 (36.70%)	809 (35.34%)		
Postgraduate	224 (9.79%)	287 (12.54%)		
Prepregnancy BMI	22.23 ± 3.51	22.13 ± 3.31	−1.03	0.300
Family history of diabetes	92 (4.02%)	105 (4.59%)	0.90	0.344
Parity	0.32 ± 0.54	0.42 ± 0.58	5.76	< 0.001
Gravidity	1.98 ± 1.25	2.13 ± 1.37	3.85	< 0.001
History of cesarean section	459 (20.05%)	513 (22.41%)	3.81	0.051
History of abnormal pregnancy	147 (6.42%)	8 (0.35%)	129.02	< 0.001
ART	275 (12.01%)	215 (9.39%)	8.23	0.004
PIH	64 (2.80%)	10 (0.44%)	40.05	< 0.001
GTH	161 (7.03%)	65 (2.84%)	35.94	< 0.001
Hypothyroidism in pregnancy	29 (1.27%)	12 (0.52%)	7.11	0.008
Thalassemia pregnancy	30 (1.31%)	17 (0.74%)	3.63	0.057
PCOS	27 (1.18%)	7 (0.31%)	11.85	0.001
HT	yyn (0.57%)	8 (0.35%)	1.20	0.274

Abbreviations: AAM, age at menarche; ART, assisted reproductive technology; GTH, gestational transient hyperthyroidism; HT, Hashimoto′s thyroiditis; PCOS, polycystic ovary syndrome; PIH, pregnancy‐induced hypertension.

**Figure 2:**
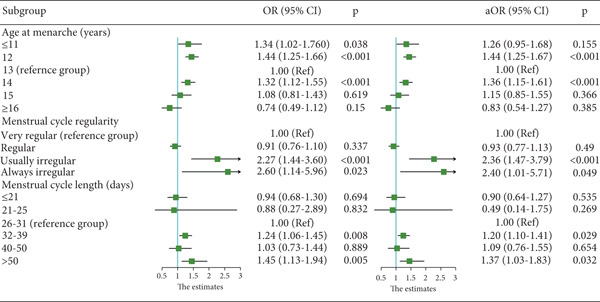
Forest plots illustrating the associations of AAM and menstrual cycle characteristics with GDM risk, both before and after adjustment for confounders.

Figure 2 presents the ORs derived from a conditional multivariate logistic regression analysis for GDM across different AAM groups and menstrual characteristics. The OR values represent estimates that are unadjusted for confounding variables, whereas the aOR values reflect estimates that have been adjusted for confounders. Specifically, adjustments were made for education, parity, gravidity, history of abnormal pregnancy, ART, PIH, PCOS, GTH, and hypothyroidism during pregnancy.

Menarche at or before 11 years was significantly associated with an increased risk of GDM analysis (OR: 1.34, 95% CI: 1.02–1.76, *p* = 0.038). However, after additional confounders were adjusted, the relationship was not significant. In contrast, menarche at 12 years continued to be a highly significant predictor of GDM risk, regardless of whether confounding factors were adjusted for, and was associated with an approximately 44% increase in risk (OR = 1.44, 95% CI: 1.25–1.66, *p* < 0.001; aOR = 1.44, 95% CI: 1.25–1.67, *p* < 0.001). Compared with the reference group (menarche at 13 years), the group who experienced menarche at 14 years of age was also associated with a significantly elevated risk of GDM (aOR = 1.36, 95% CI: 1.15–1.61, *p* < 0.001). In contrast, the risk for GDM in the menarche‐at‐15‐year group did not differ significantly from the reference value. Moreover, there was no difference in the risk for GDM in the menarche‐at‐16‐years‐or‐later group.

For assessment of menstrual cycle regularity on GDM risk, the most regular category (cycles varying by only 3–4 days) served as the reference group. Women who reported having “usually irregular” cycles had an OR of 2.27 (95% CI: 1.44–3.60, *p* < 0.001), indicating that their risk of developing GDM was approximately 127% than that of women in the reference group. After adjustment for confounders, the association between usually irregular menstrual cycles and GDM risk persisted and even amplified, with an aOR of 2.36 (95% CI: 1.47–3.79, *p* < 0.001). Women reporting usually irregular menstrual cycles exhibited a more than twofold increased risk of GDM compared to those with regular cycles. A similar pattern was observed among women with “always irregular” cycles. Analysis of GDM in relation to menstrual cycle regularity yielded an OR of 2.60 (95% CI: 1.14–5.96, *p* = 0.023), which corresponds to an approximately 160% increase in the odds of developing GDM compared with the reference group. Furthermore, this effect of “always irregular” cycles remained statistically significant after adjusting for confounders (aOR = 2.40, 95% CI: 1.01–5.71, *p* = 0.049), indicating that even after the analysis adjusted for potential confounders, irregular cycles were significantly associated with an elevated risk of GDM.

Compared with the 26–31‐day reference range, longer‐than‐average menstrual cycles (32–39 days and especially those cycles exceeding 50 days) were associated with a significantly greater likelihood of GDM. In contrast, shorter cycles (< 21 or 21–25 days) did not significantly increase the risk. These findings underscore the need to closely monitor women who present with notably longer menstrual cycles, given their elevated risk of developing GDM.

**Figure 3:**
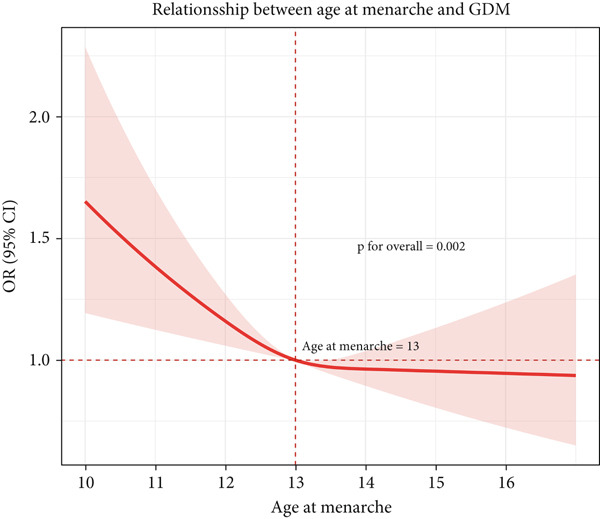
Restricted cubic spline analysis illustrates the association between AAM and the risk of GDM. The solid lines represent the ORs, whereas the shaded red area indicates the 95% CI.

The restricted cubic spline analysis revealed a nonlinear relationship between AAM and the odds of GDM (*p* < 0.05) (see Figure 3) after adjusting for education, parity, gravidity, history of abnormal pregnancy, ART, PIH, PCOS, GTH, and hypothyroidism during pregnancy. On the horizontal axis, AAM is measured in years, whereas the vertical axis depicts the OR for GDM on a logarithmic scale. Notably, the spline‐based estimate (represented by the solid line) intersects the OR value of 1.0 at approximately 13 years, thereby designating this age as the reference point. For AAM < 13 years, the risk curve exceeds 1.0, and the 95% CI does not cross 1, indicating a statistically significantly elevated risk of GDM among women with early menarche (defined as AAM < 13 years). Beyond the 13‐year threshold, the association curve levels off, with the 95% CI crossing 1, indicating that there is no statistically significant relationship between AAM and GDM risk in this range.

## 4. Discussion

In this investigation, we conducted an exploratory study involving 4578 pregnant women, including 2289 with GDM and 2289 normal control subjects. Given that PCOS is a well‐established cause of both menstrual cycle irregularity and insulin resistance [29], we adjusted for PCOS status in our multivariate analysis. Although age and BMI are also important determinants, age was controlled for by design through matching [30], and BMI was not included in the model due to its potential role as a mediator rather than a confounder. After controlling for key risk factors, we found that a significant association between an earlier AAM and irregular and prolonged menstrual cycles remained with an increased risk of GDM. Our study employed statistical methodologies, including restricted cubic spline transformations and logistic regression inflection point analyses, to ascertain the presence of a nonlinear relationship between AAM and the occurrence of GDM. Notably, our results revealed a nonlinear association characterized by an L‐shaped curve, which indicated that the relationship between AAM and the risk of GDM was particularly pronounced at specific thresholds. The primary findings—that an earlier AAM, irregular menses, and particularly long cycle lengths are associated with an elevated risk of GDM—are robust. As the incidence of GDM continues to increase, an earlier onset of menarche may represent an important contributing factor.

AAM is a significant milestone marking the onset of sexual maturity and an important indicator of female endocrine development during childhood [31]. Our findings indicated that women who experienced menarche at either 12 or 14 years demonstrated a higher risk of developing GDM. In the unadjusted analysis, women who experienced menarche before age 11 exhibited a significantly higher risk of developing GDM. However, once we controlled for potential confounders—including PCOS—the association between AAM (< 11 years) and GDM risk was no longer statistically significant. Evidence indicates that an unusually early onset of menarche is linked to PCOS [32]. PCOS, a metabolic disorder, serves both as a potential consequence of early menarche and an independent risk factor for GDM. After adjusting for these metabolic abnormalities, the observed association between premature menarche (AAM < 11 years) and GDM risk may be attenuated or eliminated.

Several studies have examined the relationship between AAM and GDM, with findings consistently indicating a strong association between the two. Several primary studies outlined below corroborate our findings; however, another study reported that the association between AAM and GDM was not statistically significant among American women [13]. Lu et al. [15], employing Mendelian randomization analysis, demonstrated that genetic polymorphisms predisposing to early menarche are independently associated with an increased risk of GDM, which is consistent with our findings. Chen et al. proposed that an earlier‐than‐normal AAM (< 11 years) was significantly correlated with an elevated risk of GDM, on the basis of data from the Nurses′ Health Study II [33]. Furthermore, the study indicates that excessive prepregnancy adiposity is largely responsible for this association. Notably, Petry et al. reported that the relationship between AAM and GDM risk followed a U‐shaped pattern in a longitudinal cohort study, and insulin resistance potentially mediated this association [34]. The underlying mechanisms by which early menarche elevates the risk of GDM remain unclear. AAM is influenced primarily by a combination of intrinsic and extrinsic factors. Earlier AAM has been associated with higher estrogen levels and a decreased binding capacity of sex hormones—attributable to low globulin levels in adulthood—while elevated blood levels of estradiol and testosterone and/or reduced sex hormone–binding globulin have been linked to an increased risk of GDM [33, 35]. In addition, dietary habits and life stressors during childhood, such as familial conflict, divorce, and abuse, are common, modifiable factors associated with early menarche and the occurrence of GDM [36, 37]. Furthermore, the association between AAM and GDM may facilitate the identification of females at an elevated risk of developing GDM and thereby support the adoption of early intervention strategies for prevention. Further research is necessary to investigate the effects of modifiable childhood factors, with a focus on preventing premature menarche.

Menstrual cycle patterns serve as key indicators of women′s health [38]. During the reproductive years, menstrual dysfunction is commonly marked by extended or irregular cycles. In the present study, we demonstrated that menstrual characteristics were strongly correlated with the risk of GDM. The results of the conditional logistic regression analysis indicated that cycle length was positively associated with an increased risk of GDM for cycles of 32–39 days and for cycles > 50 days or too irregular to estimate. Similarly, irregular menstrual cycles were found to be associated with an elevated risk of GDM. Specifically, women with consistently irregular menstrual cycles had a 1.40‐fold increased risk of developing GDM. These associations remain independent of prepregnancy BMI and other well‐established risk factors for GDM, including age and PCOS.

The available evidence on the association between menstrual cycle characteristics and GDM is sparse and inconsistent. Our findings align with those from the Nurses′ Health Study II, which revealed that participants with extended or highly erratic menstrual cycles were more likely to develop GDM [39]. These observations suggest that a shift from healthy to unhealthy menstrual cycle patterns may be correlated with insulin resistance, a critical determinant in the development of GDM. In a large‐scale longitudinal study involving 2046 American women, Soria‐Contreras et al. discovered that women with prolonged or disrupted menstrual cycles had increased odds of developing GDM [40], whereas another study revealed that irregular menstrual cycles were not associated with GDM in South Indian women [41]. Due to potential methodological limitations, inconsistencies in study findings have emerged. By leveraging a larger sample and comprehensive data on key covariates—such as BMI and preexisting medical conditions—our study advances previous research. We also found that the link between long, irregular menstrual cycles and an increased risk of GDM remained significant after adjusting for prepregnancy PCOS. Although the association between prolonged and irregular menstrual cycles and the risk of GDM is attenuated, it remains present. This finding indicates that these associations are not solely due to PCOS or other common gynecological conditions. An irregular and prolonged menstrual cycle may serve as a marker of adverse metabolic phenotypes. An altered hormonal balance is posited to be a crucial factor linking menstrual cycle irregularities with the occurrence of GDM. One proposed mechanism of endocrine disruption suggests that the synergistic interaction with pituitary gonadotropins elicits androgen production in ovarian theca cells, thereby further impairing insulin sensitivity and leading to hyperglycemia, thereby contributing to an increased incidence of GDM [18, 42].

Current management of women at risk for GDM often underutilizes easily obtainable reproductive and menstrual indicators, which may limit early identification of high‐risk individuals and delay the implementation of preventive interventions. This study is consistent with previous findings [39], confirming that earlier AAM, irregular menstrual cycles, and prolonged cycle length are significantly associated with an increased risk of GDM. As noninvasive, easily obtainable, and cost‐effective indicators, AAM and menstrual characteristics can be incorporated into routine preconception counseling and prenatal care. Informing women with earlier menarche about their potential health risks may facilitate the adoption of healthier lifestyle behaviors at an earlier stage, thereby reducing their future risk of developing GDM. Encouraging the integration of these indicators into risk stratification frameworks may enable the early identification of women at high risk for GDM, thereby facilitating the delivery of tailored health education, lifestyle interventions, and personalized prenatal care to improve maternal metabolic health and pregnancy outcomes.

This study offers several notable strengths. First, it was one of the few investigations to explore the relationship between AAM, menstrual cycle characteristics, and GDM. Moreover, by matching cases and controls based on maternal age, we minimized the influence of individual susceptibility and confounding factors. In addition, we collected detailed data on demographics, lifestyle factors, and medical histories for all participants and employed conditional logistic regression to adjust for potential confounders and verify the robustness of our findings. However, several limitations of our study should be acknowledged. First, a margin of error inevitably exists in self‐reported data regarding menstrual characteristics. Second, we were unable to secure sufficient data on the use of medications, such as oral contraceptives, which influence menstrual cycle characteristics and are frequently prescribed for the management of menstrual disorders. Third, as participants were recruited exclusively from a single center, the universality of our findings may be limited.

## 5. Conclusions

In conclusion, our study revealed a positive correlation between AAM, prepregnancy menstrual characteristics, and the risk of GDM, even after adjusting for potential confounding variables. An L‐shaped association between AAM and GDM was observed, with a cut‐off age of 13 years used as the reference. These findings suggest that health care professionals should consider AAM and menstrual cycle characteristics as important indicators for assessing patients′ metabolic risk. However, additional research is needed to confirm the potential pathophysiological mechanisms linking AAM and menstrual characteristics with GDM, which may facilitate a deeper understanding of female pubertal development and endocrine function. Such insights could inform the development of targeted lifestyle interventions to mitigate the adverse effects of menstrual disorders on pancreatic islet function.

**Nomenclature**
AAM: age at menarche
GDM: gestational diabetes mellitus
IADPSG: International Association of Diabetes and Pregnancy Study Groups
BMI: body mass index
ART: assisted reproductive technology
GTH: gestational transient hyperthyroidism
PCOS: polycystic ovary syndrome
PIH: pregnancy‐induced hypertension
HT: Hashimoto′s thyroiditis

## Ethics Statement

All procedures performed in studies involving human participants were in accordance with the 1964 Helsinki Declaration and its later amendments or comparable ethical standards. This retrospective study was approved by the Ethics Committee of the Wuhan Union Hospital of Huazhong University of Science and Technology (No. TJ‐IRB20220611). The requirement for informed consent was waived by the ethics committee because the study involved the analysis of anonymized retrospective data, posed no more than minimal risk to participants. All data were anonymized to ensure confidentiality and comply with ethical standards. The date of the first trial registration was 2022.09.01. The Ethics Committee of Union Hospital, Tongji Medical College, Huazhong University of Science and Technology, approved the waiver of informed consent for this study.

## Consent

The authors have nothing to report.

## Conflicts of Interest

The authors declare no conflicts of interest.

## Author Contributions

Conceptualization of topic: all authors. Data collection: C.X., J.H., and H.W. Software: J.H. and Y.S. Formal analysis: Y.S., J.H., Q.Y., C.X., H.W., R.L., and J.Z. Writing—original draft: J.H. and Y.S. Supervision: Q.Y. and J.M. Validation: R.L. and J.Z. Review and editing: R.L., J.Z., J.M., and J.H. Funding acquisition: H.W. Y.S. and Q.Y. contributed equally to this study as first authors.

## Funding

The study was funded by the Wuhan Nursing Association in China, WHHL202201.

## Data Availability

The datasets utilized and analyzed in the present study are accessible from the corresponding authors upon reasonable request.
